# Erzhi Tiangui Granules Improve In Vitro Fertilization Outcomes in Infertile Women with Advanced Age

**DOI:** 10.1155/2021/9951491

**Published:** 2021-08-12

**Authors:** Jinlong Sun, Jing-Yan Song, Yao Dong, Shan Xiang, Qiong Guo

**Affiliations:** ^1^Reproductive and Genetic Center of Integrated Traditional and Western Medicine, The Affiliated Hospital of Shandong University of Traditional Chinese Medicine, Jinan 250014, Shandong, China; ^2^The First Clinical College, Shandong University of Traditional Chinese Medicine, Jinan 250014, Shandong, China; ^3^Women's Health Department, Maternal and Child Health Care Hospital of Shandong Province, Jinan 250011, Shandong, China

## Abstract

**Background:**

The fertility of females with advanced age declines with aging. Therefore, for medical and social reasons, it is important to establish mechanisms to protect and improve the fertility of such populations. With widespread use of traditional Chinese medicine (TCM) in in vitro fertilization (IVF), studies have evaluated their impact on improving the fertility of females with advanced age. In this study, we performed proteomic analysis of follicular fluid to reveal mechanisms of the Erzhi Tiangui (EZTG) granule (Chinese herbs for replenishing vital essence to tonify the kidney) in improving the outcomes of IVF in infertile women with advanced age.

**Methods:**

This was a randomized, double-blind, and placebo-controlled trial in which 100 patients with advanced age were divided into the EZTG group and the placebo group by the random number table plus envelope method. Both groups were subjected to controlled ovarian stimulation with a GnRH antagonist regimen. Differences between the two groups were evaluated, including the TCM syndrome score after treatment, gonadotrophin (Gn) days and Gn doses, the number of retrieved oocytes, 2 pronucleus (PN) fertilization, 2PN cleavage, and high-quality embryos. Differentially expressed proteins were identified using the LC-MS/MS method, and their functions were determined through bioinformatics analyses.

**Results:**

The number of high-quality embryos in the placebo group was significantly lower than that in the EZTG group (2.88 ± 1.85 vs. 4.13 ± 2.83, *p*=0.011). Eleven differentially expressed proteins were identified between the two groups. Four proteins were highly expressed, whereas seven were suppressed in the control group, compared to the EZTG group. The overall trend suggested that the apoptotic effect in the follicular fluid of the EZTG group was downregulated.

**Conclusion:**

Treatment with the EZTG granule can improve embryo quality in IVF of advanced age females with both kidney Qi and Yin deficiency syndromes. The mechanism is attributed to downregulation of apoptotic-effector protein expressions in the follicular fluid. This trial is registered with ChiCTR1900025139.

## 1. Introduction

The threshold for female reproductive aging is ≥35 years [1–3]. Due to increasing late marriages and childbearing (especially since October 2015), the proportion of pregnancy among women with advanced age has further increased, specifically with the “two-child policy” in China. Since fertility decreases with age [4, 5], older women with reduced or lost fertility often seek help from assisted reproductive technologies (ART). There are several adjuvant treatment strategies for infertile advanced age women undergoing IVF, including coenzyme Q10 [6], dehydroepiandrosterone (DHEA) [7], growth hormone (GH) [8], and recombinant luteinizing hormone (r-LH) [9]. However, it has not been determined whether these drugs can improve pregnancy outcomes in advanced age women, especially older women with decreased ovarian reserves.

Kidney-tonifying TCM plays a universal role in delaying human life aging and prolonging the human reproductive period [10–12]. By evaluating the efficacy and mechanism of kidney-tonifying TCM in ART superovulation, we found that kidney-tonifying can improve oocyte quality through a variety of pathways and improve normal fertilization rates, cleavage rates, and pregnancy rates of in vitro fertilization-embryo transfer in patients with kidney deficiency syndrome [13].

Studies involving TCM are based on two mechanisms: “holistic concept” and “syndrome differentiation and treatment.” The decline in female fertility involves multisystem, multiway, and multilink changes of the body, including proteomics. Proteomics, as a component of system biology, reflects the holistic concept of TCM and shows the advantages and roles of TCM in promoting fertility in women with advanced age. Therefore, we performed proteomic analysis of the follicular fluid to establish the mechanisms of Erzhi Tiangui (EZTG) granule (a TCM for replenishing vital essence to tonify the kidney) in improving IVF outcomes in infertile women with advanced age. Our findings provide a scientific, effective, and systematic theory and elucidate on the method of TCM for protecting the fertility of such populations.

## 2. Materials and Methods

### 2.1. Patient Enrollment and Grouping

This study is registered in the Chinese Clinical Trial Registry (ChiCTR1900025139). The 100 infertile patients were recruited from the Reproductive and Genetic Center of Integrated Traditional and Western Medicine, the Affiliated Hospital of Shandong University of Traditional Chinese Medicine, Jinan, China (PRC) from June 2015 to December 2016. Eligible participants were infertile patients undergoing IVF/intracytoplasmic sperm injection (ICSI) procedures without contraindications for adverse IVF/ICSI treatment outcomes.

Participant enrollment was performed by staff not involved in the randomization process. A computer-based random number generator was used for grouping, according to the random number allocation sequence. Women with advanced age were allocated into two groups based on computer-assisted block randomization. Sequence generation and assignment of participants to the experimental and control groups were made by a study staff member who was not involved in intervention delivery, data collection, or data analysis. Both medications (Erzhi Tiangui formula and placebo) were prepared, so that they were identical in shape, taste, and smell. The experimental group (*n* = 50) was orally administered with Erzhi Tiangui, while the control group (*n* = 50) was administered with a placebo.

### 2.2. Criteria for Syndrome Differentiation for Qi and Yin Kidney Deficiencies

- Primary symptoms are as follows: (i) light menstrual color and thin texture, (ii) fatigue, (iii) lumbosacral soreness, (iv) light red tongue with dental marks and a thin white or less coating, and (v) both chi pulses exhibit a deep thready pulse or a deep thready and rapid pulse.
- Secondary syndromes are as follows: (i) dizziness and tinnitus, (ii) dry mouth and throat, (iii) dry vagina, and (iv) lower leg ache or talalgia.

The kidney Qi and Yin deficiency syndrome was diagnosed only by the presence of all primary syndromes with one or two secondary syndromes.

### 2.3. Inclusion and Exclusion Criteria

Married women aged between 35 and 44 years old, receiving autologous oocytes and who had kidney Qi and Yin deficiency syndromes were enrolled in the study. Patients diagnosed with premature ovarian insufficiency and who had body mass index (BMI) of ≥30 kg/m^2^, endometriosis, polycystic ovarian syndrome, severe malformation of reproductive organs, major operation history, and had used hormonal drugs within 3 months before the study were excluded. A couple with karyotyping abnormalities was also excluded.

### 2.4. Preparation of the Erzhi Tiangui Granule and the Placebo

The Drug Manufacturing Unit of the Affiliated Hospital of Shandong University of Traditional Chinese Medicine produced the EZTG granule. The EZTG was packaged as 3 g/bag, batch number 01-FZ032-03. The daily dose is equivalent to 15 g of *Ligustrum lucidum* (Nv Zhen Zi), 15 g of *Lotus japonicus* (Han Lian Cao), 15 g of the fruit of Chinese wolfberry (Gou Qi Zi), 15 g of *Cuscuta chinensis* (Tu Si Zi), 15 g of Radix Rehmanniae Preparata (Shu Di Huang), 12 g of *Angelica sinensis* (Dang Gui), 12 g of *Paeonia lactiflora* (Bai Shao), 12 g of *Ligusticum wallichii* (Chuan Xiong), 12 g of *Rhizoma cyperi* (Xiang Fu), and 9 g of Radix Glycyrrhizae Preparata (Zhi Gan Cao). The placebo granule, which was mainly composed of dextrin, was made in a similar color and shape to EZTG. Placebo granules were packaged as 3 g/bag, with the same package of the EZTG, batch number 01-FZ032-03-1. The EZTG or placebo was orally administered after being dissolved in water, 3 g each time, 2 times a day.

### 2.5. In Vitro Fertilization and Sample Collection

After the 2nd to 3rd day of menstruation and 150–300 units of exogenous gonadotropin controlled ovarian stimulation, the EZTG group was administered with Erzhi Tiangui granules, while placebo granules were administered to the placebo group for 11–14 days. After vaginal ultrasound confirmation of the follicle diameter of between 18 and 20 mm, participants were administered with a single dose of 10000 units of HCG (human chorionic gonadotropin) as a “trigger.” Then, 36 h after HCG injection, oocyte retrieval and extraction of ovarian granulosa cells were conducted under transvaginal ultrasound.

The follicular fluid was obtained after oocyte retrieval, and the presence of a cumulus complex was confirmed by inverted microscopy. After centrifugation for 10 min at 3000 rpm to separate red blood cells, leucocytes, and follicle cells, the supernatant was recovered in Eppendorf (EP) tubes, labeled and refrigerated at −80°C for further examination.

### 2.6. Sample Preparation

Briefly, XX of protein was supplemented with 50 mM ammonium bicarbonate to YY, and DTT was added to a final concentration of 10 mM for 60 min at 37°C. Iodoacetamide was added to the solution to a final concentration of 50 mM for 30 min at room temperatures in the dark. The solution was transferred to the ultrafiltration tube and centrifuged for 10 min at 14,000 rpm. Pellets were rinsed twice using 50 mM ammonium bicarbonate (containing 0.8% SDC). Trypsin was added after which samples were enzymatically hydrolyzed under incubation at 37°C for 12–16 h. Then, the enzymatic hydrolysates were rinsed using 50 mM ammonium bicarbonate and merged, followed by TFA acidification shaking, centrifugation to remove SDC, and desalting with a C18 desalting column. Desalted samples were lyophilized in a freeze-dryer and redissolved in 0.1% FA for mass spectrometry detection.

### 2.7. Liquid Chromatography-Mass Spectrometry (LC-MS/MS) Analysis

Desalting of 100 mg lyophilized TMT-labeled peptide pools was performed on a 100 mg C18 solid-phase extraction column Sep-Pak (Waters, Wilmslow, UK) and further fractionated using either reverse-phase chromatography combined with elution at a high pH, isoelectric focusing on an Agilent 3100 OFFGEL fractionator (Agilent, Santa Clara, CA, USA) or HILIC chromatography. Each time, 18–24 fractions were collected and analyzed using a nanoflow LC-MS/MS. Nanoflow LC-MS/MS was performed on an 1100 series capillary LC system (Agilent) coupled with an LTQ-Orbitrap mass spectrometer (Thermo Scientific) operating in the positive mode and equipped with a nanoarray source. Peptide mixtures were trapped in a ReproSil C18 reverse-phase column (column dimensions: 1.5 cm × 100 *μ*m, packed in-house; Dr. Maisch GmbH, Ammerbuch-Entringen, Germany) at a flow rate of 8 *μ*L/min. Peptide separation was performed on the ReproSil C18 reverse-phase column (column dimensions: 15 cm × 50 *μ*m, packed in-house; Dr. Maisch GmbH) using a linear gradient from 0 to 80% B (*A* = 0.1% formic acid; *B* = 80% (v/v) acetonitrile, 0.1% formic acid) in 70 min and at a constant flow rate of 200 nL/min using a splitter. Column eluent was directly sprayed into the ESI source of the mass spectrometer. Mass spectra were acquired in a continuum mode and peptide fragmentation performed in a data-dependent mode.

### 2.8. Database Retrieval and MaxQuant Analysis

Data retrieval from databases was performed using the Proteome Discoverer software (Version PD1.4, Thermo Scientific, city, USA). Protein data were retrieved from the UniProt database (owner, city, country), with the maximum deviation of parent ion molecular weight not exceeding 10 ppm and the maximum deviation of daughter ion molecular weight not exceeding 0.02 Da. Peptide false discovery rate (FDR) was set to <1%. For each group, the experiment was repeated thrice using the MaxQuant software (Version 1.4.0.8, owner(s), city, country). We used “uniprot_Proteomes-Human.fasta” to search the original files. After configuring database files, 12 samples of original files were imported into the analysis software. Corresponding label-free quantification parameters were set and imported into the database for retrieval. The retrieved results were screened using the strict criteria (peptide FDR ≤1% and protein FDR ≤1%). For proteins to be used for quantification, they were to have ≥2 characteristic polypeptides.

### 2.9. Statistical Analysis

Statistical analysis was performed using the SPSS 19.0 statistical software (SPSS Inc., Chicago, IL, USA). *P* < 0.05 was considered statistically significant. Quantitative data for each group were expressed as mean ± SD. The data were not statistically described using the median and quartile deviation. Comparison of means between two groups was performed using Student's *t*-test. Proportions were compared using the chi-square test.

## 3. Results

### 3.1. Patient Enrollment

**Figure 1:**
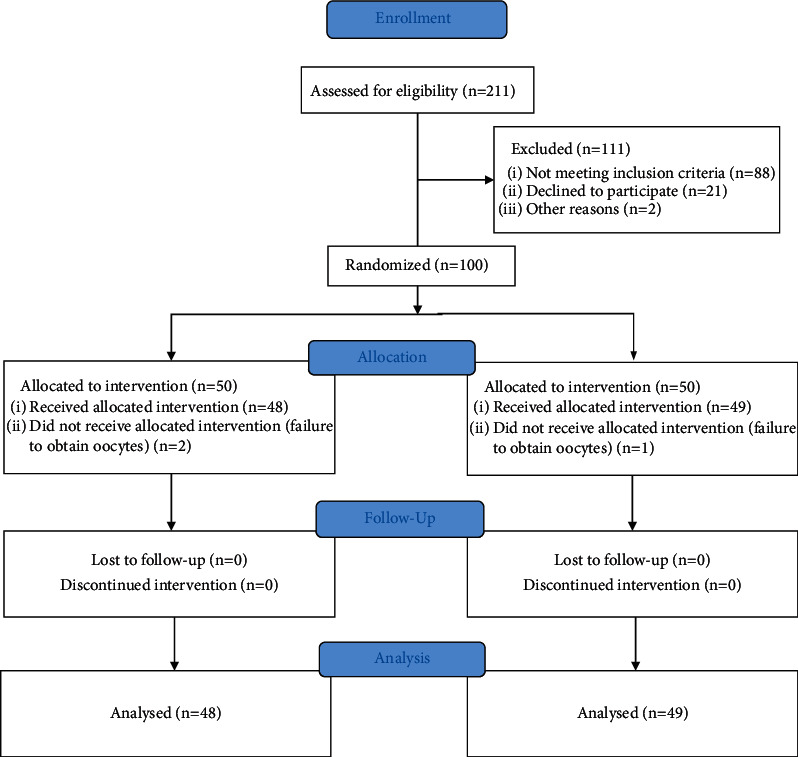
CONSORT 2010 flow diagram.

After controlled ovarian stimulation, 3 patients canceled the IVF cycles due to failure to obtain oocytes, including two in the EZTG group and one in the placebo group. The remaining 97 women completed the follow-up without major protocol violations, and all were included in outcome analyses (Figure 1). Compared to the placebo group, the EZTG group did not exhibit statistical differences in all characteristics except for the number of high-quality embryos, which was high (2.88 ± 1.85 vs. 4.13 ± 2.83, *p*=0.011) (Table 1).

### 3.2. Quantitative Analysis of Proteins

The LFQ values from MaxQuant analysis were used to characterize protein abundance. The LFQ value of the total protein for each sample was corrected. Differential proteins between EZTG and control groups were screened using a protein abundance ratio of >1.67 and <0.6. A small number of proteins that were relatively specific in the follicular fluid and played a localized role was reduced to >1.1 and <0.9. After comparisons, 11 differentially expressed proteins between the placebo and EZTG groups were identified. Four proteins were highly expressed, whereas seven were suppressed in the placebo group (Table 2).

### 3.3. Biological Function Analysis of Differentially Expressed Proteins

The differentially expressed proteins were found to be involved in physiological processes, such as lipid metabolism, immunity, cell differentiation, proliferation, and apoptosis. Some of these proteins may be involved in several different biological function processes (Table 3).

### 3.4. Safety Observation in Clinical Trials

Patients in the EZTG group and the placebo group had no adverse reactions during the medication period.

## 4. Discussion

Acceleration of apoptotic effects in elderly women results in a decline in egg and embryo quality. Abnormal growth and development of follicles and accelerated apoptosis of granulosa cells lead to follicular atresia and are the fundamental causes of the decline in ovarian reserve functions and fertility in elderly women [14].

Many of the differential proteins found in this study are involved in apoptotic and proliferative processes. They include matrix metalloproteinases (MMPs), which are crucial in apoptosis, and metalloproteinase inhibitor-1 (TIMP-1), which binds MMPs to suppress their biological activities. In addition, TIMP-1 directly binds cell surface receptors to promote the proliferation of fibroblasts, epithelial cells, smooth muscle cells, and lymphocytes [15, 16]. Antiapoptotic effects of TIMP-1 are associated with its direct inhibition of caspase 3 activity [17].

Insulin-like growth factor binding protein-3 (IGFBP-3) and IGFBP-6 compete with IGF receptors to bind IGF, thereby blocking IGF receptor formation and inhibiting IGF activity. Moreover, IGFBP-3 has an independent role in promoting apoptosis [18]. The insulin-like growth factor binding protein complex acid-labile subunit (IGFALS) binds free IGFs to form heterotrimers, which may significantly prolong the half-life of IGFs and enhance the effects of IGFs in promoting cell proliferation and inhibiting cell apoptosis [19, 20].

Transforming growth factor beta-inducible protein ig-h3 (TGF*β* Ip) is important in cell adhesion, migration, proliferation, and differentiation [21]. Retinol-binding protein 4 (RBP4), a new adipocytokine, participates in the pathological process of human insulin resistance and is associated with the lipid metabolism [22]. RBP4 has been shown to inhibit the first polar body excretion of porcine oocytes. The expressions of GDF-9 and BMP-15 in porcine oocytes treated with RBP4 interventions were found to be significantly suppressed, implying that these proteins might be involved in the apoptotic process [23]. Coiled-coil domain protein (CCDC) is an important regulator of the cell division cycle, and downregulation of CCDC can lead to decreased cyclin A expression, which means cell cycle arrest. Flow cytometry revealed that the proportion of cells in the G1 phase increased, whereas the proportion of cells in S phase and G2/M phase decreased after downregulation of CCDC expression [24]. Comparing the changes in follicular fluid protein expressions between the EZTG and the placebo groups, we found that EZTG prescription, a TCM for tonifying kidney and nourishing Yin, inhibited the apoptosis of cells in follicles to a certain extent and promoted cell proliferation, differentiation, and repair.

The pregnancy rate in IVF-ET cycles is mainly determined by the quality of embryos transferred and the receptivity of the endometrium to embryos, especially the former [25–27]. We found that the number of high-quality embryos per oocyte retrieval cycle was significantly increased in the EZTG group when compared to the placebo group. This significant change in clinical outcomes can improve the chances of successful clinical pregnancy.

According to TCM, women naturally have the essence of kidney deficiency after their “Qi Qi” age. During implementation of modern ART, multiple ovarian follicles are induced to simultaneously develop with gonadotropins. As the human body synchronously develops and matures a large number of follicles in a short period, the kidney secretes a large amount of skull and kidney essence to promote transient exuberance of kidney Qi. This may be the reason as to why syndrome scores of the two groups after treatment were lower than before treatment, with no significant differences between groups. Behind this phenomenon must be the loss of kidney Qi and kidney Yin (kidney essence), which enhances the essence of kidney deficiency in elderly women. The classic saying “Yang transforms Qi and Yin forms.” Yin shaping refers to the process by which Yin and Qi dominate the static state, and the invisible substance is formed into a tangible substance in the movement of “calming.” The “shaping” process involves follicle's self-development, maturation, and ovulation and increase in follicle volume, follicular fluid, and proliferation of granulosa cells. They are the functional aspects of kidney Yin. The process of follicular development and shaping is inseparable from cell proliferation. Therefore, the function of kidney Yin “shaping” predominates the whole process of follicular development.

Lian, using a kidney invigoration and Yin nourishment technique, formulated the EZTG prescription [28, 29]. The EZTG prescription replenishes vital essence to tonify kidneys, nourishing blood, and regulating Chong. During follicular development, especially the development of multiple follicles, abundant kidney Yin and decayed water from the human body is required. However, kidney Yin and decayed water cannot be autogenously indefinite. This could be amended by the EZTG prescription, which mobilizes human kidney functions, increases kidney Yin and decayed water reserves, and provides the products needed for the development of multiple follicles through targeted kidney tonification and Yin replenishment. This “reproductive kidney” of the ovary can be excreted to promote the maturation and ovulation of multiple follicles. Normal ovulation functions can greatly improve the chances of conception, the so-called “Jing Tiao Zi Si.”

In this study, identification of TCM syndromes was carefully performed by experienced clinicians. Due to individual differences among patients, symptomatic severity was different. The overall dose of TCM granules cannot be adjusted. Therefore, the inability of the total dose of EZTG granules to adjust according to symptomatic severity is a limitation of this study. Moreover, we did not determine any side effects associated with the EZTG granules. In conclusion, the EZTG granule had a positive effect on advanced age women with both kidney Qi and Yin deficiency syndromes.

## 5. Conclusions

The EZTG prescription intervention can lead to differential expression of some apoptosis-related proteins in the granulosa cell follicular fluid. These proteins should be quantitatively analyzed to establish the specific mechanism involved in replenishing vital essence to tonify kidneys during ovarian granulosa cell apoptosis and their role in assisted reproduction.

## Figures and Tables

**Table 1:** Characteristics of study participants.

Parameter	EZTG group	Placebo group	*P* value
Patients	48	49	—
Age (years)	38.92 ± 4.43	37.61 ± 4.22	0.139
BMI (kg/m^2^)	25.42 ± 5.73	24.05 ± 4.33	0.187
AMH (ng/ml)	1.75 ± 0.52	1.90 ± 0.41	0.118
Baseline day 3 FSH (mIU/ml)	10.53 ± 1.72	10.00 ± 2.16	0.185
Baseline day 3 E_2_ (pg/ml)	49.70 ± 11.83	53.50 ± 11.20	0.108
E_2_ on HCG day (pg/ml)	4258.75 ± 1135.11	4509.83 ± 1249.35	0.303
P on HCG day (ng/ml)	1.29 ± 0.26	1.39 ± 0.33	0.101
TCM clinical syndrome score	13.24 ± 2.17	14.60 ± 3.52	0.155
Gn days (d)	9.26 ± 2.28	9.78 ± 2.79	0.318
Gn doses (U)	3450.75 ± 1237.55	3625.85 ± 1803.25	0.579
Retrieved oocytes (*n*)	8.13 ± 3.22	7.85 ± 2.55	0.636
No. of 2PN fertilization	6.91 ± 2.87	5.87 ± 2.66	0.067
No. of 2PN cleavage	6.11 ± 2.13	5.39 ± 2.74	0.152
No. of good quality embryos	4.13 ± 2.83	2.88 ± 1.85	**0.011**
Transferred embryos per cycle	1.75 ± 0.53	1.80 ± 0.50	0.634
Cumulative pregnancy rate	32.4% (36/111)	28.7% (27/94)	0.566

AMH, anti-Müllerian hormone; FSH, follicle-stimulating hormone; E_2_, estradiol; P, progesterone; TCM, traditional Chinese medicine; Gn, gonadotropin. *P* < 0.05 indicates a statistically significant difference between the 2 groups of data. The value in bold indicates that the number of high-quality embryos was significantly increased in the EZTG group compared with the placebo group.

**Table 2:** Identification of differentially expressed proteins in follicular fluids of the EZTG group and placebo group.

Protein IDs	Protein names	Gene names	Abundance ratio	Up/down
P02753	Retinol-binding protein 4	RBP4	1.86	Up
P08637	Low affinity immunoglobulin gamma Fc region receptor III-A	FCGR3A	1.69	Up
P24592	Insulin-like growth factor binding protein-6	IGFBP-6	1.17	Up
P17936	Insulin-like growth factor binding protein-3	IGFBP-3	1.13	Up
P01033	Metalloproteinase inhibitor-1	TIMP-1	0.87	Down
Q15582	Transforming growth factor beta-induced protein ig-h3	TGFBI	0.82	Down
P19438-5	Isoform 5 of tumor necrosis factor receptor superfamily member 1A	TNFRSF1A	0.71	Down
P35858	Insulin-like growth factor binding protein complex acid-labile subunit	IGFALS	0.7	Down
Q12789-3	Isoform 2 of the general transcription factor 3C polypeptide 1	GTF3C1	0.63	Down
Q8NEF3	Coiled-coil domain-containing protein 112	CCDC112	0.6	Down
Q07864	DNA polymerase epsilon catalytic subunit A	POLE	0.56	Down

Abundance ratio, EZTG group/placebo group.

**Table 3:** Biological function analysis for differentially expressed proteins between EZTG and placebo groups.

Biological function	Identified differential proteins
Lipid metabolism	Retinol-binding, protein 4 (RBP4)
Immunization	Low affinity immunoglobulin gamma Fc region receptor III-A (FCGR3A)
Cell differentiation, proliferation and apoptosis	Retinol-binding protein 4 (RBP4), metalloproteinase inhibitor-1 (TIMP-1), insulin-like growth factor binding protein-3 (IGFBP-3), isoform 5 of the tumor necrosis factor receptor superfamily member 1A (TNFRSF1A), insulin-like growth factor binding protein-6 (IGFBP-6), insulin-like growth factor binding protein complex acid-labile subunit (IGFALS), DNA polymerase epsilon catalytic subunit A (POLE), isoform 2 of general transcription factor 3C polypeptide 1 (GTF3C1), transforming growth factor beta-induced protein ig-h3 (TGFBI), and coiled-coil domain-containing protein 112 (CCDC112)

## Data Availability

The data used to support the findings of this study are available from the corresponding author upon request.

## Additional Points

*Highlights. *(1) What do we know about this topic? In China, Chinese herbs for replenishing vital essence to tonify the kidney can improve in vitro fertilization outcomes in infertile women with advanced age. However, their specific roles and internal mechanisms have not been clearly evaluated. (2) How does our research contribute to this field? From the perspective of follicular fluid proteomics, we established the mechanism involved in replenishing vital essence to tonify the kidneys in order to improve in vitro fertilization outcomes in infertile women with advanced age. (3) What are the implications of our research in theory, practice, or policy? Chinese herbs for replenishing vital essence to tonify the kidney should be popularized and applied to benefit infertile women with advanced age.

## References

[1] Usta I. M., Nassar A. H. (2008). Advanced maternal age. Part I: obstetric complications. *American Journal of Perinatology*.

[2] Kimberly L., Case A., Cheung A. P. (2012). Advanced reproductive age and fertility: no. 269, November 2011. *International Journal of Gynaecology and Obstetrics: The Official Organ of the International Federation of Gynaecology and Obstetrics*.

[3] Jiang L., Chen Y., Wang Q. (2019). A Chinese practice guideline of the assisted reproductive technology strategies for women with advanced age. *Journal of Evidence-Based Medicine*.

[4] Klein J., Sauer M. V. (2001). Assessing fertility in women of advanced reproductive age. *American Journal of Obstetrics and Gynecology*.

[5] Johnson J.-A., Tough S., Wilson R. D. (2012). Delayed child-bearing. *Journal of Obstetrics and Gynaecology Canada*.

[6] Xu Y., Nisenblat V., Lu C. (2018). Pretreatment with coenzyme Q10 improves ovarian response and embryo quality in low-prognosis young women with decreased ovarian reserve: a randomized controlled trial. *Reproductive Biology and Endocrinology*.

[7] Nagels H. E., Rishworth J. R., Siristatidis C. S., Kroon B. (2015). Androgens (dehydroepiandrosterone or testosterone) for women undergoing assisted reproduction. *Cochrane Database of Systematic Reviews*.

[8] Norman R. J., Alvino H., Hull L. M. (2019). Human growth hormone for poor responders: a randomized placebo-controlled trial provides no evidence for improved live birth rate. *Reproductive BioMedicine Online*.

[9] Humaidan P., Chin W., Rogoff D. (2017). Efficacy and safety of follitropin alfa/lutropin alfa in ART: a randomized controlled trial in poor ovarian responders. *Human Reproduction*.

[10] Shen M., Qi C. (2017). Effect of bushen jianpi formula on reproductive ability and egg quality of in vitro fertilization of natural aging mice. *Journal of Traditional Chinese Medicine*.

[11] Liancheng G., Yujia C., Mengzhi Z., Zongling W., Huimin R. (2019). Mechanism of anti-aging by tonifying kidney herbs. *Liaoning Journal of Traditional Chinese Medicine*.

[12] Li W., Aiping L. (2018). Kidney essence and TCM delaying senility. *Modern Journal of Integrated Traditional Chinese and Western Medicine*.

[13] Lian F., Wu H.-C., Sun Z.-G., Guo Y., Shi L., Xue M.-Y. (2014). Effects of Liuwei Dihuang Granule (六味地黄颗粒) on the outcomes of in vitro fertilization pre-embryo transfer in infertility women with Kidney-yin deficiency syndrome and the proteome expressions in the follicular fluid. *Chinese Journal of Integrative Medicine*.

[14] Jessberger R. (2012). Age‐related aneuploidy through cohesion exhaustion. *EMBO Reports*.

[15] Pang I.-H., Hellberg P. E., Fleenor D. L., Jacobson N., Clark A. F. (2003). Expression of matrix metalloproteinases and their inhibitors in human trabecular meshwork cells. *Investigative Opthalmology & Visual Science*.

[16] Seo D.-W., Li H., Guedez L. (2003). TIMP-2 mediated inhibition of angiogenesis. *Cell*.

[17] Maitra S. R., Bhaduri S., El-Maghrabi M. R., Shapiro M. J. (2005). Inhibition of matrix metalloproteinase on hepatic transforming growth factor *β*1 and caspase-3 activation in hemorrhage. *Academic Emergency Medicine*.

[18] Price D., Muterspaugh R., Clegg B. (2020). IGFBP-3 blocks hyaluronan-CD44 signaling, leading to increased acetylcholinesterase levels in A549 cell media and apoptosis in a p53-dependent manner. *Scientific Reports*.

[19] Sirotkin A. V., Benčo A., Tandlmajerová A. (2018). cAMP response element-binding protein 1 controls porcine ovarian cell proliferation, apoptosis, and FSH and insulin-like growth factor 1 response. *Reproduction, Fertility and Development*.

[20] Lee C.-C., Chen P.-H., Ho K.-H. (2017). The microRNA-302b-inhibited insulin-like growth factor-binding protein 2 signaling pathway induces glioma cell apoptosis by targeting nuclear factor IA. *PLoS One*.

[21] Runager K., García-Castellanos R., Valnickova Z. (2009). Purification, crystallization and preliminary X-ray diffraction of wild-type and mutant recombinant human transforming growth factor beta-induced protein (TGFBIp). *Acta crystallographica. Section F, Structural biology and crystallization communications*.

[22] Quadro L., Hamberger L., Colantuoni V., Gottesman M. E., Blaner W. S. (2003). Understanding the physiological role of retinol-binding protein in vitamin A metabolism using transgenic and knockout mouse models. *Molecular Aspects of Medicine*.

[23] Marantidis A., Laliotis G. P., Avdi M. (2016). Association of RBP4 genotype with phenotypic reproductive traits of sows. *Genetics research international*.

[24] Derewenda U., Tarricone C., Choi W. C. (2007). The structure of the coiled-coil domain of Ndel1 and the basis of its interaction with Lis1, the causal protein of Miller-Dieker lissencephaly. *Structure*.

[25] Hsu M.-I., Mayer J., Aronshon M. (1999). Embryo implantation in *in vitro* fertilization and intracytoplasmic sperm injection: impact of cleavage status, morphology grade, and number of embryos transferred. *Fertility and Sterility*.

[26] Yaron Y., Botchan A., Amit A., Kogosowski A., Yovel I., Lessing J. B. (1993). Endometrial receptivity: the age-related decline in pregnancy rates and the effect of ovarian function. *Fertility and Sterility*.

[27] Navot D., Bergh R. A., Williams M. A. (1991). Poor oocyte quality rather than implantation failure as a cause of age-related decline in female fertility. *The Lancet*.

[28] Sun Z.-G., Lian F., Jia Q. (2012). Effects of *Er’zhi Tiangui* granule (二至天癸颗粒) on sequential expressions of integrin *β*3 and its ligand osteopontin in mouse endometrium during controlled ovarian hyperstimulation. *Chinese Journal of Integrative Medicine*.

[29] Fang L., Rui-Xia W., Feng-Mei M., Zhen-Gao S., Li-Hong W., Lei S. (2013). Effects of Chinese medicines for tonifying the kidney on DNMT1 protein expression in endometrium of infertile women during implantation period. *Journal of Alternative & Complementary Medicine*.

